# Inhibitory effect of chemical constituents from *Artemisia scoparia* Waldst. et Kit. on triglyceride accumulation in 3T3-L1 cells and nitric oxide production in RAW 264.7 cells

**DOI:** 10.1007/s11418-013-0799-3

**Published:** 2013-10-20

**Authors:** Tadahiro Yahagi, Naoyuki Yakura, Keiichi Matsuzaki, Susumu Kitanaka

**Affiliations:** School of Pharmacy, Nihon University, 7-7-1 Narashinodai, Funabashi, Chiba 274-8555 Japan

**Keywords:** *Artemisia scoparia* Waldst. et Kit., Chromane, Triglyceride, Nitric oxide

## Abstract

We investigated the anti-obesity effect of the aerial part of *Artemisia scoparia* Waldst. et Kit. (Compositae). An 80 % aqueous EtOH extract of the aerial part inhibited triglyceride (TG) accumulation and the nitric oxide (NO) production activity. A new chromane derivative was isolated from the aerial part of *A. scoparia* Waldst. et Kit. along with 18 known compounds. The structure of the new chromane, scopariachromane (**1**), was elucidated by spectroscopic analyses. The inhibitory effects of the compounds on TG accumulation activity were examined. Among these, cirsiliol (**11**) inhibited TG accumulation in 3T3-L1 preadipocytes. Jaceosidin (**12**) inhibited NO production in a murine macrophage-like cell line (RAW 264.7). These results indicate that the 80 % aqueous EtOH extract and compounds isolated from the aerial part of *A. scoparia* Waldst. et Kit. may improve obesity-related insulin resistance.

## Introduction

Obesity is one of the leading metabolic diseases worldwide [1] and is closely associated with coronary heart disease, hypertension, type 2 diabetes mellitus, cancer, respiratory complications, and osteoarthritis [2]. Obesity is a condition in which adipocytes accumulate a large amount of fat and become enlarged. At the cellular level, this condition is characterized by an increase in the number and size of adipocytes that differentiate from 3T3-L1 preadipocytes in the adipose tissue [3]. Recent studies have demonstrated that the obese adipose tissue is characterized by enhanced macrophage infiltration [4]. Macrophages produce various inflammatory proteins such as tumor necrosis factor alpha (TNF-α), monocyte chemoattractant protein-1 (MCP-1), and nitric oxide (NO), which are implicated in insulin resistance and metabolic disorders [5]. NO is a diffusible, liposoluble, free radical gas produced from l-arginine by a family of enzymes known as inducible NO synthases (iNOS) [6]. In a co-culture system of RAW 264.7 macrophages and 3T3-L1 adipocytes, marked increases in the secretion of inflammatory mediators such as TNF-α, MCP-1, and NO were observed [7]. Triglyceride (TG) is synthesized from glucose and fatty acid that is incorporated by glucose transporter 4 and fatty acid transporter (CD36) into 3T3-L1 preadipocytes [8, 9]. Cultured 3T3-L1 adipocytes have many properties similar to those of normal adipocytes. Thus, this cell line is a suitable model system for obesity-related research [10–12]. Herbal extracts from plants such as *Blumea balsamifera* [13], *Ginkgo biloba* [14], *Wasabia japonica* [15], *Zizyphus jujuba* [16], *Morus alba* var. *multicaulis* [17], and *Albizia julibrissin* Durazz [18] have been shown to possess anti-obesity effects. Thus, we aimed to screen for crude drugs and natural products with inhibitory effects on TG accumulation in adipocytes and NO production in activated macrophages.

*Artemisia scoparia* Waldst. et Kit. belongs to the family Compositae and is native to Japan, Korea, and Mongolia. The aerial part of this plant is used in traditional medicine as an antiphlogistic, as a diuretic, for the treatment of hepatitis and urticaria, and as an antimold agent. Phytochemical investigations of the aerial part of *A. scoparia* Waldst. et Kit. resulted in the isolation of flavonoids, coumarins, and essential oils [19].

In the present study, we found that an 80 % aqueous EtOH extract of *A. scoparia* Waldst. et Kit. inhibited TG accumulation [22.5 % (30 μg/mL)] in 3T3-L1 adipocytes and NO production [24.3 % (30 μg/mL)] by RAW 264.7, which were activated by lipopolysaccharide (LPS) and recombinant mouse interferon gamma (IFN-γ).

Therefore, we analyzed *A. scoparia* Waldst. et Kit. to identify the active compounds in the extract. Through bioactivity-guided fractionation, we identified a new chromane derivative, scopariachromane (**1**), and 18 known compounds from the aerial part of *A. scoparia* Waldst. et Kit. Herein, we describe the structural elucidation and biological evaluation of these compounds.

## Results and discussion

**Fig. 1:**
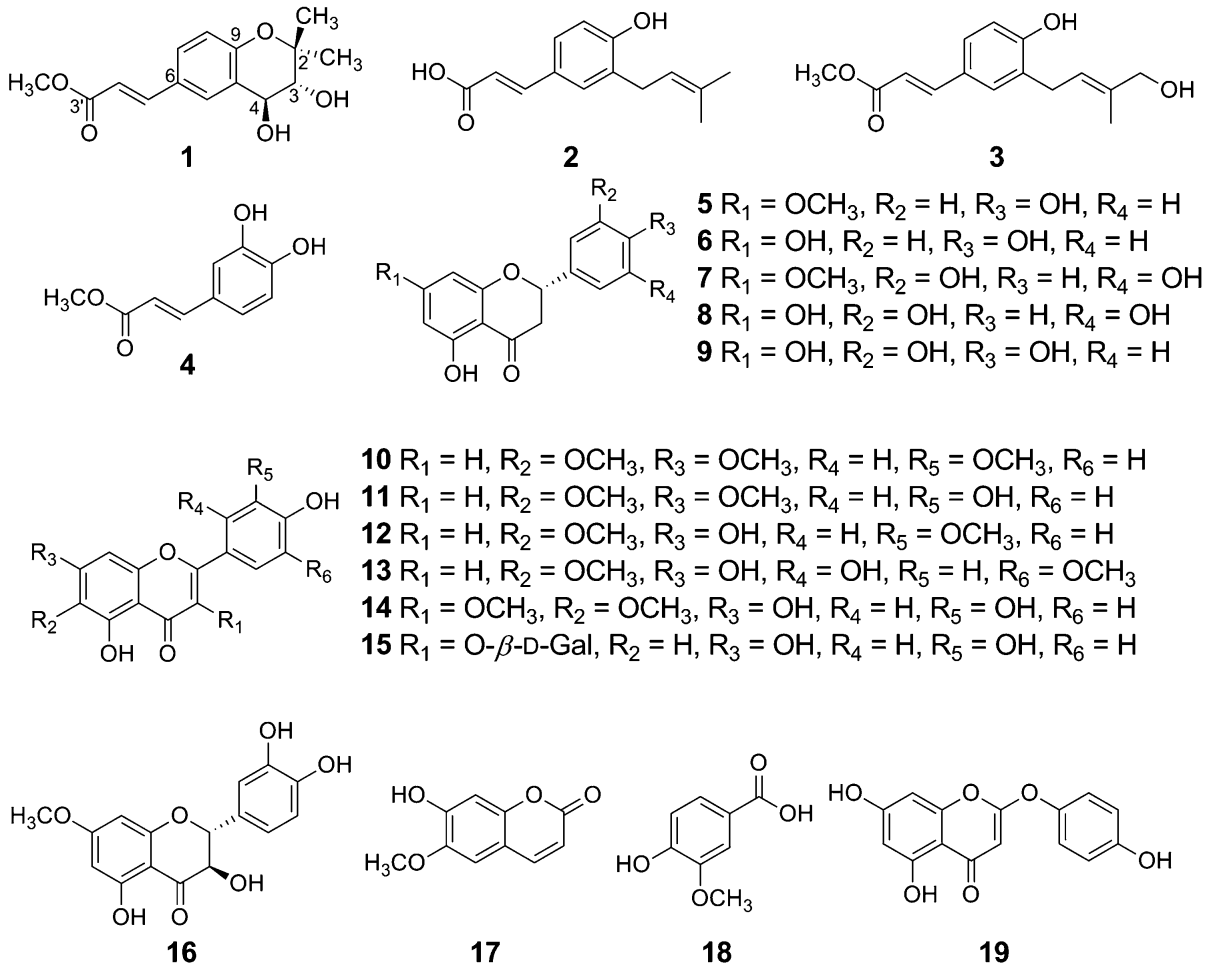
Structures of compounds isolated from *Artemisia scoparia* Waldst. et Kit

The aerial parts of *A*. *scoparia* Waldst. et Kit. were extracted with 80 % aqueous EtOH. The 80 % aqueous EtOH extract was suspended in H_2_O and partitioned with *n*-hexane, CHCl_3_, EtOAc, and *n*-BuOH, successively. The CHCl_3_ and EtOAc layers showed inhibitory effects on TG accumulation [41.9 and 10.4 %, respectively (30 μg/mL)] and NO production [55.4 and 33.1 %, respectively (10 μg/mL)]. Bioassay-directed fractionation led to the isolation of a new chromane derivative, named scopariachromane, together with 18 known compounds (**2–19**) (Fig. 1).

**Fig. 2:**
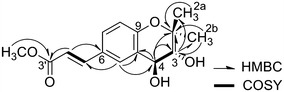
Key COSY and HMBC correlations for **1**

**Fig. 3:**
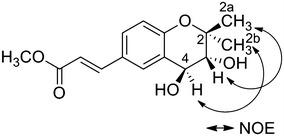
Key NOE correlations for **1**

Scopariachromane (**1**) was isolated as an amorphous colorless powder, and its molecular formula was determined as C_15_H_18_O_5_ by HR-EI-MS, with seven degrees of unsaturation. The UV spectrum of **1** showed absorption at 295 and 232 nm, and its IR spectrum showed the characteristic absorption bands of hydroxyl groups (3420 cm^−1^), a conjugated carbonyl group (1694 cm^−1^), and conjugated double bonds (1633 and 1609 cm^−1^). The ^1^H NMR chemical shifts of **1** (Table 1) indicated the presence of a 1,2,4-trisubstituted aromatic ring at *δ*_H_ 7.74 (1H, d, *J* = 1.8 Hz, H-5), *δ*_H_ 7.47 (1H, dd, *J* = 8.7, 1.8 Hz, H-7), and *δ*_H_ 6.76 (1H, d, *J* = 8.7 Hz, H-8); a* trans*-olefin at *δ*_H_ 7.60 (1H, d, *J* = 15.9 Hz, H-1′) and *δ*_H_ 6.36 (1H, d, *J* = 15.9 Hz, H-2′); a methoxy proton at *δ*_H_ 3.72 (3H, s, OCH_3_-3′); and two methyl at *δ*_H_ 1.45 (3H, s, H-2a) and *δ*_H_ 1.22 (3H, s, H-2b). Analysis of the ^13^C NMR (Table 1) and DEPT spectra revealed the presence of one carbonyl group, one methoxy group, two methyls, seven methines, and five quaternary carbons. All protonated carbons were assigned by HMQC analysis. Furthermore, the ^1^H–^1^H COSY (Fig. 2) spectrum indicated connectivity between methine protons at *δ*_H_ 4.55 (H-4) and *δ*_H_ 3.56 (H-3), H-1′ and H-2′, respectively. HMBC analysis of **1** (Fig. 2) showed long-range correlations between H-4 and *δ*_C_ 79.8 (C-2) and *δ*_C_ 75.9 (C-3); H-3 and C-2, *δ*_C_ 27.1 (C-2a) and *δ*_C_ 19.7 (C-2b); H-2b and C-2, and C-3; and H-2a and C-2, and C-3, which indicated the presence of a prenyl-type unit. Furthermore, H-5 and *δ*_C_ 126.1 (C-10) and *δ*_C_ 69.1 (C-4); H-4 and *δ*_C_ 129.2 (C-5) and C-10; and H-3 and C-10 indicated that the prenyl-type unit was located on C-4. The degree of unsaturation at seven also indicated that the prenyl-type unit participated in a cyclic structure. The NOESY spectrum of **1** (Fig. 3) showed correlations between H-2a and H-3, and H-2b and H-4. These results suggested a* quasi-trans axial*. The coupling constant between H-3 and H-4 (*J* = 8.7 Hz) indicated that these protons were* trans diaxial*. This analysis and a comparison with published data indicated that **1** was a (3R*,4S*)-3,4-dihydroxy-2,2-dimethyl-chroman derivative [20]. In addition, HMBC correlations between H-5 and C-1′; H-2′ and *δ*_C_ 167.2 (C-3′); H-2′ and *δ*_C_ 127.1 (C-6); and *δ*_H_ 3.72 (OCH_3_-3′) and C-3′ were observed. Thus, the relative structure of **1** was determined to be scopariachromane.

**Table 1:** ^1^H (300 MHz) and^13^C NMR (75 MHz) data for compound **1** (acetone-*d*_6_ with TMS as the internal standard)

Position	**1**	
	*δ*_C_	*δ*_H_
2	79.8	
2a	27.1	1.45 (3H, s)
2b	19.7	1.22 (3H, s)
3	75.9	3.56 (1H, d, *J* = 8.7)
4	69.1	4.55 (1H, d, *J* = 8.7)
5	129.2	7.74 (1H, d, *J* = 1.8)
6	127.1	
7	129.0	7.47 (1H, d, *J* = 8.7, 1.8)
8	117.7	6.76 (1H, d, *J* = 8.7)
9	154.9	
10	126.1	
1′	144.8	7.60 (1H, d, *J* = 15.9)
2′	115.4	6.36 (1H, d, *J* = 15.9)
3′	167.2	
OCH_3_	54.4	3.72 (3H, s)

Chemical shifts are given in ppm; coupling constants *J* (in parentheses) are given in Hz

The known isolated compounds were readily identified by comparison of physical and spectral data with published values. These compounds included three *p*-coumaric acid derivatives, drupanin (**2**) [21], methyl 3-[4′-hydroxyprenyl]coumarate (**3**) [22], and methyl 1-(3′,4′-dihydroxyphenyl)propenoate (**4**) [23]; five flavanes, sakuranetin (**5**) [24], naringenin (**6**) [25], blumeatin (**7**) [26], 3′,5,5′,7-tetrahydroxyflavanone (**8**) [26], and eriodictyol (**9**) [27]; four flavones, cirsilineol (**10**) [28], cirsiliol (**11**) [28], jaceosidin (**12**) [29], and 5,7,2′,4′-tetrahydroxy-6,5′-dimethoxyflavone (**13**) [30]; two flavonols, axillarin (**14**) [31] and hyperin (**15**) [32]; a flavanonol, 7-methoxytaxifolin (**16**) [33]; a coumarin, scopoletin (**17**) [34]; a benzoic acid derivative, vanillic acid (**18**) [35]; and a chromone derivative, 6-demethoxycapillarisin (**19**) [36]. Compounds **2**, **3**, **5**–**9**, **13**, **14**–**16**, and **18** were isolated for the first time from the aerial part of *A. scoparia* Waldst. et Kit.

The isolated compounds were evaluated for their inhibitory effects against intracellular TG accumulation in 3T3-L1 cells. To determine their nontoxic concentrations, 3T3-L1 cells were treated with various concentrations (3–100 μM) of **1**–**19**, and the cell viability was measured by 3-(4,5-dimethylthiazol-2-yl)-2,5-diphenyltetrazolium bromide (MTT) assay. None of the compounds were cytotoxic at concentrations up to 30 μM. Thus, we used a concentration of at most 30 μM. TG measurements were corrected for the amount of DNA. Quercetin, which has been reported to have inhibitory effects on TG accumulation, was used as a positive control [37]. Treatment with **7**, **10**, **11**, and **16** (30 μM) inhibited TG accumulation (Table 2). In particular, **11** potently inhibited intracellular TG accumulation. Compared with **10**, **11** showed a stronger inhibitory effect on TG accumulation. These results suggest that the presence of a hydroxy group at C-3′ could be important. We also examined the effects of **7**, **10**, **11**, and **16** on the uptake of [1-^3^H]-2-deoxy-d-glucose (Table 3). TG is synthesized from glucose and fatty acid that are incorporated by glucose transporter 4 and fatty acid transporter CD36 [8, 38]. These compounds inhibited glucose uptake for TG synthesis in 3T3-L1 adipocytes. In addition, we have examined the inhibitory effects of **1**–**19** on NO production in RAW 264.7 cells. As shown in Table 4, compounds **1**–**14**, **16**, **17**, and **19** showed inhibitory activity. These compounds did not have cytotoxic effects (10–30 μM). In the assay, aminoguanidine (IC_50_ 17.5 μM), which has been reported to have inhibitory effects on NO production in LPS-activated RAW 264.7 macrophages via the downregulation of iNOS, was used as a positive control [39]. Compounds **4**, **5**, **7**–**11**, **12**–**14**, **16**, and **17** showed strong or moderate inhibitory effects on NO production compared with aminoguanidine (Table 4). Of the 19 compounds tested, compound **12** exhibited the highest inhibitory activity against NO production (IC_50_ 5.9 μM).

**Table 2:** Effects of compounds **1**–**19** on TG accumulation in cultured 3T3-L1 adipocytes

	Inhibition (%)		Inhibition (%)
**1**	5.9 ± 3.0	**11**	77.0 ± 0.9**
**2**	7.1 ± 3.2	**12**	20.1 ± 14.3
**3**	−1.5 ± 5.0	**13**	4.0 ± 3.0
**4**	5.7 ± 7.1	**14**	19.1 ± 0.9
**5**	13.2 ± 3.6	**15**	−10.6 ± 4.2
**6**	2.6 ± 11.0	**16**	49.8 ± 9.6*
**7**	38.9 ± 3.1*	**17**	18.5 ± 1.3
**8**	−2.4 ± 4.0	**18**	−37.8 ± 6.1
**9**	−10.5 ± 3.0	**19**	−8.8 ± 3.6
**10**	37.2 ± 3.7*	Quercetin	38.4 ± 3.4

Cells were treated with compounds **1**–**19** (30 μM) on days 0 and 3. On day 8, the intracellular TG content of the cells was measured

Results are expressed as mean ± SE of 3 individual experiments

*p < 0.05, **p < 0.01 vs. control (Student’s *t* test)

**Table 3:** Effect of compounds **7**, **10**, **11**, and **16** on glucose uptake in 3T3-L1 cells

	Inhibition (%)		Inhibition (%)
**7**	44.4 ± 3.2*	**16**	19.8 ± 3.2
**10**	67.4 ± 1.3**	Quercetin	20.3 ± 1.7
**11**	63.1 ± 0.2**		

After cell differentiation, the medium was replaced with serum-free DMEM for 2 h, and the cells were washed and incubated in KRH buffer containing insulin (200 nM) for 10 min. After exposure to insulin, they were treated with samples in KRH buffer for 15 min. After incubation, [1-^3^H]-2-deoxy-d-glucose (0.25 μCi/mL) was added, and incubation was continued for 10 min. The cells were solubilized in 0.1 % SDS. The incorporated radioactivity was measured by liquid scintillation counting

Results are expressed as the mean ± SE of 3 individual experiments

*p < 0.05, **p < 0.01 vs. control (Student’s *t* test)

**Table 4:** Inhibitory effects of the isolated compounds **1**–**19** on NO production stimulated by LPS and IFN-γ in RAW 264.7 cells

	IC50 (μM)		IC50 (μM)
**1**	73.7	**11**	18.0
**2**	46.4	**12**	5.9
**3**	41.2	**13**	13.0
**4**	21.1	**14**	23.1
**5**	25.1	**15**	>100
**6**	55.2	**16**	13.7
**7**	20.6	**17**	21.1
**8**	25.7	**18**	>100
**9**	27.3	**19**	38.2
**10**	13.1	Aminoguanidine	17.5

In brief, the results presented here showed that an 80 % aqueous EtOH extract of the aerial part of *A. scoparia* Waldst. et Kit. and the isolated compounds may be used to reduce obesity, which is a serious health problem in industrialized countries, by inhibiting NO production by activated macrophages, and intracellular TG accumulation and glucose uptake in mature adipocytes.

## Experimental

### General experimental procedure

Optical rotation (OR) was measured in MeOH using a JASCO P-1020 polarimeter. The UV spectra were obtained in MeOH using a JASCO V-550 spectrophotometer, and the IR spectra were recorded using a JASCO IR A-2 spectrophotometer. The NMR spectra were recorded using a JEOL ECX-500 spectrometer (^1^H NMR, 500 MHz; ^13^C NMR, 125 MHz), with TMS as an internal standard. Mass spectra were obtained using a JEOL GCmate spectrometer. Silica gel 60 N (Kanto Chemical Corp.), YMC GEL ODS-A (YMC Co. Ltd.), and Sephadex LH-20 (GE Healthcare) were used for column chromatography (CC). TLC was performed using TLC plates (thickness 0.25 mm, F254; Merck), and compounds were visualized by spraying with 5 % (v/v) H_2_SO_4_ in EtOH and vanillin reagent. HPLC was performed using a JASCO PU-1580 apparatus equipped with a JASCO UV-1575 detector and a Shodex OR-2 OR detector. Cosmosil 5C18-MS-II (10 × 250 mm i. d. and 4.6 × 250 mm i. d.; Nacalai Tesque), Cosmosil Cholester (10 × 250 mm i. d.; Nacalai Tesque), and Cosmosil *π* nap (10 × 250 mm i. d.; Nacalai tesque) were used for preparative purposes.

### Plant materials

The aerial part of *A*. *scoparia* Waldst. et Kit. was collected from Inner Mongolia during 2003. An authentic specimen of this plant was deposited in the Laboratory of Pharmacognosy, School of Pharmacy, Nihon University, Japan (NK-03040).

### Extraction and isolation

The aerial part of *Artemisia scoparia* Waldst. et Kit. (5 kg) was extracted 3 times with 80 % aqueous EtOH. Evaporation of the solvent under reduced pressure from the combined extract afforded the EtOH extract (265 g). The extract was suspended in H_2_O (1:1, v/v) and partitioned with *n*-hexane (4 × 1:1, v/v), CHCl_3_ (4 × 1:1, v/v), EtOAc (4 × 1:1, v/v), and *n*-BuOH (4 × 1:1, v/v), successively. The amounts extracted were 0.543, 4.43, 30.0, and 56.6 g, respectively, and the residual aqueous extract yielded 135.1 g of material. The CHCl_3_ extract was subjected to silica gel CC, [*n*-hexane/EtOAc (100:0 → 0:100, v/v)] to yield Fr. C-1–C-16. Fr. C-5 (409 mg) was purified by reversed-phase HPLC with H_2_O/MeOH (70:30, v/v) to yield **5** (93.6 mg). **7** (44 mg) was crystallized from Fr. C-7 (559 mg) using MeOH. Fr. C-8 (213 mg) was purified by reversed-phase HPLC with H_2_O/MeOH (43:57, v/v) to yield **2** (2.5 mg).

Fr. C-9 (624 mg) was subjected to ODS CC [H_2_O/MeOH (90:10 → 0:100, v/v)] to yield Fr. C-9-1–C-9-5. Fr. C-9-2 (416 mg) was purified by reversed-phase HPLC with H_2_O/MeOH (65:35, v/v) to yield **3** (96 mg), and **12** (9.0 mg). Fr. C-10 was purified by reversed-phase HPLC with H_2_O/MeOH (35:65, v/v) to yield **1** (7.6 mg), **10** (4.0 mg), and **17** (8.6 mg). The EtOAc extract was subjected to silica gel CC [CHCl_3_/MeOH (100:0 → 0:100, v/v)] to yield Fr. E-1–E-5. Fr. E-2 (2463 mg) was subjected to ODS CC [H_2_O/MeOH (90:10 → 0:100, v/v)] to yield Fr. E-2-1–E-2-6. Fr. E-2-1 (329.1 mg) was subjected to Sephadex LH-20 CC [H_2_O–MeOH (50:50, v/v)] to yield Fr. E-2-1-1–E-2-1-3. Fr. 2-1-2 (28.9 mg) was purified by reversed-phase HPLC with H_2_O/MeOH (50:50, v/v) to yield **18** (7.7 mg). Fr. E-2-3 (845 mg) was purified by reversed-phase HPLC with H_2_O/CH_3_CN (60:40, v/v) to yield **4** (96 mg), **6** (29.3 mg), **8** (15.8 mg), **11** (40.7 mg), **13** (12.3 mg), **16** (39.7 mg), and **19** (12.9 mg). Fr. E-3 (2781 mg) was subjected to ODS CC [H_2_O/MeOH (90:10 → 0:100, v/v)] to yield Fr. E-3-1–E-3-5. Fr. E-3-3 (518 mg) was purified by reversed-phase HPLC with H_2_O/CH_3_CN (60:40, v/v) to yield **9** (98.1 mg). Fr. E-3-4 (701 mg) was subjected to Sephadex LH-20 CC [H_2_O/MeOH (20:80, v/v)] to yield Fr. E-3-4-1–E-3-4-12. Fr. E-3-4-7 (26.8 mg) was purified by reversed-phase HPLC with H_2_O/CH_3_CN (60:40, v/v) to yield **14** (3.8 mg). Fr. E-5 (9206.2 mg) was subjected to ODS CC [H_2_O/MeOH (90:10 → 0:100, v/v)] to yield Fr. E-5-1–E-5-7. Fr. E-5-4 (1557 mg) was purified by reversed-phase HPLC with H_2_O/CH_3_CN (10:90, v/v) to yield Fr. E-5-4-1–E-5-4-3. **15** (47.7 mg) was crystallized from Fr. E-5-4-2 (370.9 mg) using MeOH.

Scopariachromane (**1**): amorphous colorless powder. $[\alpha ]_{\text{D}}^{25}$–40.3° (*c* = 1.0, MeOH). UV *λ*_max_ (MeOH) nm (log *ε*): 215 (4.00), 232 (3.99), 295 (4.03). IR (KBr) *ν*_max_ cm^−1^: 3420 (OH), 1694 (conj. C=O), 1633 (conj. C=C). EI-MS *m*/*z*: 232 [M]^+^. HR-EI-MS *m*/*z*: 278.1153 (calcd for C_15_H_18_O_5_, 278.1154). The ^1^H and ^13^C NMR spectral data for **1** are presented in Table 1.

### Nitrite assay

The cells were seeded at 1.2 × 10^6^ cells/mL onto 96-well flat-bottom plates (Sumitomo Bakelite) and incubated at 37 °C for 2 h. The test sample was then added to the culture simultaneously with *Escherichia coli* LPS (100 ng/mL) and recombinant mouse IFN-γ (0.33 ng/mL), and the cells were incubated at 37 °C, usually for 16 h. After incubation, the cells were chilled on ice. The culture supernatant (100 μL) was placed in wells in duplicate 96-well flat-bottom plates. A standard solution of NaNO_2_ was also placed in other wells on the same plates. To quantify nitrite, 50 μL Griess reagent, 1 % sulfanilamide in 5 % H_3_PO_4_, and 0.1 % *N*-(1-naphthyl)ethylenediamide dihydrochloride were added to each well. After 10 min, the reaction products were colorimetrically quantified at 550 nm with subtraction of the background absorbance at 630 nm, using a model 3550 microplate reader (BIO-RAD).

### TG assay

3T3-L1 preadipocytes (American Type Culture Collection) were plated in 24-well plates and maintained in DMEM supplemented with 10 % (v/v) fetal calf serum (FCS) and 1 % (v/v) penicillin–streptomycin at 37 °C in a humidified 5 % CO_2_ incubator. To induce differentiation, 3-day postconfluent 3T3-L1 preadipocytes (day 0) were stimulated by adipogenic agents (500 μM 3-isobutyl-1-methylxanthine, 1 μM dexamethasone, and 10 μg/mL insulin) that were added to DMEM with 10 % (v/v) fetal bovine serum (FBS) culture medium. After 3 days, the medium was replaced with DMEM containing 10 % (v/v) FBS and 5 μg/mL insulin, and it was subsequently replaced every 3 days. The cells were harvested 8 days after the initiation of differentiation. The cells were washed with PBS (−), scraped on ice in 500 μL of sonication buffer (25 mM Tris buffer and 1 mM EDTA; pH 7.5), and sonicated to homogenize the cell suspension. The total TG content of the cells was determined using the LabAssay™ triglyceride kit (Wako Pure Chemical Industries, Ltd., Osaka, Japan). The DNA concentration was determined using the DNA quantity kit (Primary Cell Co., Ltd., Sapporo, Japan). The TG concentration per microgram of DNA in 3T3-L1 cells was expressed as the ratio (%) relative to the control value. The test sample dissolved in DMSO was added.

### Glucose uptake assay

3T3-L1 adipocytes were harvested 8 days after the initiation of differentiation. After the differentiation, the medium was replaced with serum-free DMEM for 2 h, and the cells were then washed and incubated in KRH buffer (25 mM HEPES, 120 mM NaCl, 5 mM KCl, 1.2 mM KH_2_PO_4_, 1 mM CaCl_2_, 1 mM MgSO_4_; pH 7.4) containing insulin (200 nM) for 10 min at 37 °C. After exposure to insulin, the cells were washed and treated with samples in KRH buffer for 15 min. After incubation, [1-^3^H]-2-deoxy-d-glucose (0.25 μCi/mL) was added, and incubation was continued for 10 min. The cells were washed twice with ice-cold KRH buffer and then solubilized with 0.1 % SDS. The incorporated radioactivity was measured by liquid scintillation counting. Nonspecific uptake was determined in the presence of 20 μM cytochalasin B and was subtracted from the total value. The test sample dissolved in DMSO was added.

### Cell viability assay

Cell viability was assessed using MTT. The 3T3-L1 cells (1.0 × 10^5^ cells/mL) were seeded in 96-well plates and incubated for 24 h at 5 % CO_2_ and 37 °C and then treated with samples. After 24 h of incubation, 20 μL of MTT solution (1 mg/mL) was added to the cell culture, and the cells were further incubated at 37 °C and 5 % CO_2_ for 4 h. After removing the medium, the MTT formazan crystals were dissolved in DMSO, following which the absorbance in individual wells was determined at 570 nm using a microplate reader and the background absorbance (655 nm) was subtracted. The test sample dissolved in DMSO was added.
